# Novel Poly(Vinylidene Fluoride)/Montmorillonite Polymer Inclusion Membrane: Application to Cr(VI) Extraction from Polluted Water

**DOI:** 10.3390/membranes11090682

**Published:** 2021-09-01

**Authors:** Ferhat Sellami, Ounissa Kebiche-Senhadji, Stéphane Marais, Charles Lanel, Kateryna Fatyeyeva

**Affiliations:** 1 Laboratoire de Procédés Membranaires et de Technique de Séparation et de Récupération (LPMSTR), Faculté de Technologie, Université de Bejaia, Bejaia 06000, Algeria; ferhat-06@live.fr (F.S.); kebiche_anissa@yahoo.fr (O.K.-S.); 2 Normandie University, UNIROUEN, INSA Rouen, CNRS, Polymères, Biopolymères, Surfaces (PBS), 76000 Rouen, France; 3 Normandie University, UNIROUEN, UFR Sciences et Technique, 76000 Rouen, France; charles.lanel@univ-rouen.fr

**Keywords:** polymer inclusion membrane, Cr(VI) removal, montmorillonite, membrane rigidity, stability

## Abstract

Novel hybrid polymer inclusion membranes (PIMs) based on poly(vinylidene fluoride) (PVDF) (polymer matrix) and Aliquat 336 (ion carrier) and containing native sodium (Cloisite Na^+^ (CNa)) and organo-modified (Cloisite 30B (C30B)) Montmorillonites were elaborated and tested for the removal of toxic Cr(VI) ions from the aqueous solution. The influence of the nanoclay incorporation on the physicochemical properties of PVDF-based PIMs was studied and the resulting membrane transport properties of the Cr(VI) ions were investigated in detail. The water contact angle measurements reveal that the incorporation of the CNa nanofiller affects the membrane wettability as less hydrophilic surface is obtained in this case—~47° in the presence of CNa as compared with ~15° for PIMs with C30B. The membrane rigidity is found to be dependent on the type and size of the used Montmorillonite. The increase of Young’s modulus is higher when CNa is incorporated in comparison with C30B. The stiffness of the PIM is strongly increased with CNa amount (four times higher with 30 wt %) which is not the case for C30B (only 1.5 times). Higher Cr(VI) permeation flux is obtained for PIMs containing CNa (~2.7 µmol/(m^2^·s)) owing to their porous structure as compared with membranes loaded with C30B and those without filler (~2 µmol/(m^2^·s) in both cases). The PIM with 20 wt % of native sodium Montmorillonite revealed satisfactory stability during five cycles of the Cr(VI) transport due to the high membrane rigidity and hydrophobicity. Much lower macromolecular chain mobility in this case allows limiting the carrier loss, thus increasing the membrane stability. On the contrary, a deterioration of the transport performance is recorded for the membrane filled with C30B and that without filler. The obtained results showed the possibility to extend the PIM lifetime through the incorporation of nanoparticles that diminish the carrier loss (Aliquat 336) from the membrane into the aqueous phase by limiting its mobility within the membrane by tortuosity effect and membrane stiffening without losing its permselective properties.

## 1. Introduction

Rapid industrialization, urbanization, and a growing boom in the development of synthetic materials in many fields of application have led to water pollution which is one of the considerate concerns to be taken care of the current world [1]. Being non-degradable in nature, heavy metals are significant pollutants of soil and aquatic ecosystems. Hexavalent chromium ion is one of the heavy metal ions with a high toxicity even at very low concentrations. It is toxic to humans, flora, and fauna [2,3]. For the treatment of wastewater containing Cr(VI) ions, several physicochemical methods are applied, such as liquid–liquid extraction [4], adsorption on activated carbons and low cost adsorbents [5], chemical and electrochemical reduction-precipitation [6] and ion exchange [7]. However, these so-called conventional methods have several significant drawbacks. For example, the adsorption is rather expensive process (i.e., the higher is the quality, the greater is the cost) with low selectivity. Besides, it has a limitation in the recollection of the adsorbent after its binding with Cr(VI) and a hazard of the secondary pollution with the adsorbent itself. The chemical precipitation is linked to the production of toxic sludge [2,8,9]. In any case, the main limitations of these methods are incomplete removal, high energy consumption, and a non-eco-friendly process. 

Green technologies and eco-friendly methodologies are in a desperate need to maintain a sustainable human society. In this context, the development of green protocols for the elimination of ionic metal species from water attracts much attention in the last years [1,10,11]. Membrane technology has undergone unprecedented development. Recently, membrane-based separation is considered to be one of the most important, efficient, and useful separation technologies in the chemical and associated process industry and, thus, it can successfully compete with conventional separation processes. Today, membranes are far and wide used to produce potable water, to treat industrial effluents and to recover valuable constituents; they are also key components in energy conversion, storage systems, chemical reactors, and in drug delivery devices. Compared to conventional procedures, membrane processes are often environmentally friendly, energy efficient, easier to operate and have the advantage of operating at ambient temperature avoiding any change or degradation of products [10,12]. The membrane market including filtration and electrodialysis has grown significantly; however, the industrial application of liquid membranes remains limited because of their insufficient stability, thus preventing their large-scale application [13,14].

Considerable efforts have been provided to improve the stability of liquid membranes, as demonstrated by the numerous works published on this topic [14,15]. The effort is paid; a new class of liquid membrane usually called polymer inclusion membranes (PIMs) has emerged [15,16,17]. PIMs are a type of liquid membranes whose extractant (carrier) is immobilized between the membrane polymer chains. In some cases, a plasticizer or modifier is added to the membrane to improve both the membrane flexibility and compatibility of membrane compounds [17,18,19].

Owing to the undoubtedly numerous features of PIMs, in particular, the use of reduced amount of extractant and organic reagents, thus minimizing the impact on the environment although ensuring high selectivity and separation as compared with the solvent extraction, the PIM system permits extraction and stripping processes to run at the same time, thus increasing the extraction efficiency. In addition, PIMs possess good mechanical properties and are easy to prepare [13,20]. Therefore, the application of PIMs covers the extraction and transport of metallic ions and small organic compounds from their aqueous solutions [13,21]. Besides, PIMs offer a higher and better stability than other types of liquid membranes, such as supported liquid membranes (SLMs), due to the fact that the liquid phase is located between the entangled polymer chains in a network of nanometer size channels, on the contrary to the liquid phase in case of SLMs, where it is held only by capillary forces within the micrometer size pores of an inert polymer membrane and, thus, it can be more easily released to the aqueous phase [13,22].

Despite the improved stability, it has been observed that prolonged use causes loss of the PIM performance by leaching their organic phase responsible for transport [20,23]. Several studies have been carried out with the aim to stabilize the PIMs by adjusting either the membrane composition (i.e., base polymer, extractant, and plasticizer/modifier) or the aqueous medium, in which the membrane is used. For example, a poly(vinylidene fluoride-*co*-hexafluoropropylene) (PVDF-HFP) and poly(ethylene glycol) dimethacrylate (PEG-DMA) semi-interpenetrating crosslinked polymer network [24] or a cellulose triacetate (CTA) and poly(butylene adipate-*co*-terephthalate) (PBAT) blend [2] used as a po-lymer base allow better entangling the carrier as the increased tortuosity and the reduced carrier loss were observed, thus providing reinforced membrane stability.

The molecular structure of the extractant and plasticizer/modifier also makes it possible to delay their leakage into the aqueous phase. It is observed that the highly lipophilic nature ensures the higher PIM stability [16,22,25,26]. The ionic strength of the aqueous solution in which the membrane is used influences the membrane mass loss; it is the lowest when the solution with the highest ionic strength is used [27,28]. 

The incorporation of inorganic nanoparticles into the polymer membrane presents a new and recent way for the PIM stabilization. Recently, the influence of nanofillers on the PIM stability and selectivity towards different ions has been revealed. For example, Kaya et al. compared the stability of two PIMs for the Cr(VI) transport [29]. The first PIM is composed of CTA as the polymer base, nitrophenyl octyl ether (NPOE) as plasticizer, and calix[4]arene as carrier and the second PIM has the same composition but is loaded with reduced graphene oxide (RGO) (1 wt %). It is shown that the PIM containing RGO exhibits an improved stability owing to its increased hydrophobicity character. Anticó et al. prepared nanoparticle-doped PIMs (NP-PIMs) for the removal of arsenate and phosphate from water [30]. Such PIMs were made of CTA, and Aliquat 336 (a quaternary ammonium salt) which interacts with the target species. Inorganic nanoparticles—such as Fe_3_O_4_, SiO_2_, and TiO_2_—and multiwalled carbon nanotubes (MWCNTs) were added to the polymer/carrier mixture. It is revealed that the presence of nanoparticles has no effect on mass loss results and only the PIM modified with MWCNTs shows enhanced stability. The observed membrane mass loss is around 20% in case of the PIM containing only Aliquat 336, while a mass loss of 8.2% is noted for PIM with MWCNTs. The authors explained the membrane improved stability by the MWCNT ability to adsorb organic compounds, in particular, Aliquat 336.

The leaching of the PIM carrier takes place because of its mobility within the membrane and, thus, depends on the membrane porosity, shape, nature and on the tortuosity of the diffusive pathways in the PIM polymer backbone [24]. Besides, it can be expected that such mobility also depends on the membrane stiffness. Therefore, it may be possible to improve the membrane stability by creating a more rigid PIM with a more tortuous environment in its polymer structure. Such a membrane may be obtained by incorporating nanoplatelets in the PIM structure.

It is well-known that the polymer barrier properties can be significantly enhanced by the inclusion of impermeable lamellar fillers, such as layered silicate clays, with a sufficient aspect ratio and ability to modify the diffusion path of the penetrating molecules. The main challenge in this case is to obtain a homogeneous dispersion and complete exfoliation of the nanoparticles in the polymer matrix for greater mechanical reinforcement and tortuosity increase, thus making the improvement of the barrier properties possible [31]. In the case of nanocomposites, it is often required to slow down the diffusion of small molecules (such as gases) through the polymers by increasing the tortuosity effect. However, this effect is undesirable in the case of PIMs, since it is more important to have high permeation flux of target molecules rather than hindering diffusion. Therefore, a trade-off between the diffusion and the PIM stability should be found. 

In the present study, novel PIMs based on PVDF as the polymer base, room temperature ionic liquid (RTIL) Aliquat 336 as ion carrier and containing different types of Montmorillonite (MMT) (i.e., inorganic particles with high aspect ratio) were prepared for the first time and characterized. The main idea is to study the influence of the nanoparticles’ nature (more or less hydrophilic) and structure (stacks of crystalline sheets more or less spaced) (i.e., native and organo-modified MMTs) on the structure and transport properties of PVDF/ionic liquid mixed membranes. Silicate minerals represent about 90% of the Earth’s crust and are, therefore, an important class of minerals forming rocks. Due to their low cost, high cation exchange capacity, excellent swelling tendency, high aspect ratio and ease of chemical modification [32,33], such inorganic fillers are intensively used in nanocomposite materials. 

The incorporation of MMT in PVDF/Aliquat 336 membranes allows obtaining material with enhanced mechanical and transport properties and increased performance, thus clearly underlying the high potential of such loaded PIMs for separation processes involving heavy metals. To our knowledge, the investigation on these composite membranes with the aim to find a relationship between the membrane structure, its mechanical properties and Cr(VI) extraction performance has never been performed yet. Therefore, this work brings new insight in the field of physicochemical behavior of new PIMs for the water treatment and especially for the Cr(VI) extraction.

The influence of the filler nature on the membrane properties was investigated by thermal, surface, and mechanical analysis. The obtained results were correlated with the membrane transport behavior. In addition, the composition of elaborated membranes was optimized in terms of the filler content and efficient removal of Cr(VI) from aqueous solutions. Particular interest was paid to the membrane stability by analyzing the PIM reuse in several cycles.

## 2. Materials and Methods 

### 2.1. Materials

PVDF powder and dimethylacetamide (DMAc) (≥99% purity) were purchased from Alfa Aesar. N-methyl-N,N,N-trioctylammonium chloride named Aliquat 336 (≥97% purity) and hydrochloric acid were received from Sigma Aldrich. 1.5-diphenylcarbazide (DPC) (≥97% HPLC purity) obtained from Fluka chemika, acetic acid (≥99.5% purity), and ammonium acetate (≥98% purity) provided by Biochem, potassium chromate K_2_CrO_4_ (99.5% purity) obtained from Acros were used without further purification. Native sodium MMT (Cloisite Na+) (CNa) and organo-modified MMT (Cloisite 30B) (C30B) were used. C30B was organically modified with methyl, tallow, bis-2-hydroxyethyl, and quaternary ammonium at a concentration of 90 meq/100 g of clay. The tallow chains of surfactant with a composition of ~65% C18; ~30% C16; ~5% C14 was supplied by BYK Additives (Germany). All water used in the work was milli-Q water (Milli-Q Water System, Millipore, resistivity of 18 MΩ⋅cm at 25 °C). 

### 2.2. Membrane Preparation 

**Figure 1:**
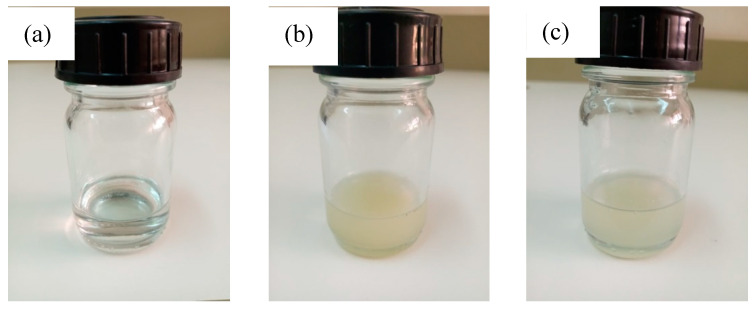
Optical images of the prepared solutions: (**a**) 70PVDF/30Aliquat 336, (**b**) 50PVDF/30Aliquat 336/20Cna, and (**c**) 50PVDF/30Aliquat 336/20C30B.

Pure PVDF- and PVDF loaded with MMT-based PIMs were prepared by casting and solvent evaporation technique. The total mass of all membrane components was kept at 1000 mg. First, a given amount of filler (CNa or C30B) was dispersed in DMAc for 30 min using the ultrasonic stirring bath, and then the polymer was added to the obtained solution—10 mL of DMAc per 1 g of polymer. The solution was left under magnetic stirring at room temperature (24 ± 1 °C) for 24 h for solubilization and formation a homogenous casting solution. Then, the carrier (i.e., Aliquat 336) was added and the final solution was additionally stirred during 24 h. In case of the PVDF/Aliquat 336 membrane, PVDF and Aliquat 336 were dissolved together. The solution containing PVDF and Aliquat 336 is transparent, whereas the solutions containing CNa and C30B nanofillers are rather opaque (Figure 1). The obtained solution was poured on a glass plate using a Doctor Blade applicator with a casting knife of 550 µm. The plate with a casted solution was then placed in an oven at 60 °C and DMAc was slowly evaporated for at least 72 h allowing a flexible and mechanically stable membrane to be formed. The average thickness of the PIMs was determined to be 40 ± 3 µm.

For clarity, the loaded PIMs are noted as XPVDF/YAliquat 336/ZNanoparticles and the values X, Y, and Z refer to the mass fraction (wt %) of PVDF, Aliquat 336 and filler, respectively.

### 2.3. Physicochemical Characterization

#### 2.3.1. Tensile Tests

Mechanical measurements were performed on an Instron 5543 machine (Norwood, MA, USA) equipped with the 500 N load cell. Tensile tests were carried out for normalized specimens (type 5A) according to ISO 527–2. The specimens were strained to the main axis at 1 mm/min at room temperature (25 ± 1 °C) and hygrometry (34 ± 4% of relative humidity). At least five samples were tested for each membrane composition.

#### 2.3.2. Water Contact Angle Measurements

The hydrophilic/hydrophobic nature of PIMs was examined by water contact angle measurements using a Multiskop goniometer (Optrel, Germany) at room temperature (25 ± 1 °C) by the sessile drop method. A water drop of 3 µL was deposited on the membrane surface. For each sample, the contact angle between the water drop and the membrane surface was determined by the CAM software and the average of 10 drops at different locations was calculated.

#### 2.3.3. Thermogravimetric Analysis (TGA)

The membrane thermal behavior was studied using the TGA analyzer (TGA Q500, TA Instruments, New Castle, DE, USA). The TGA measurements were carried out from 30 °C to 1000 °C in nitrogen atmosphere. The heating rate and the nitrogen flow rate were 10 °C/min and 90 mL/min, respectively.

#### 2.3.4. Differential Scanning Calorimetry (DSC)

DSC measurements were performed by means of the Polyma apparatus from Netzsch (Germany). The tests were performed in an aluminum pan with pierced lid in the nitrogen atmosphere from −50 °C to 200 °C at the heating rate of 10 °C/min. The results were analyzed with Proteus software.

The PVDF crystallinity degree *X_c_*_(PVDF)_ was estimated according to the following Equation (1) taking into account the polymer weight fraction.

(1) $$X_{c(\mathrm{PVDF})}=\frac{\Delta H_{m}}{\Delta {H^{0}}_{m}\left(1-\Phi \right)}\times 100$$

where Δ*H_m_* and Δ*H*^0^*_m_* are the melting enthalpy of PVDF-based PIM and theoretical 100% crystalline PVDF, respectively. *ϕ* is the total mass fraction of Aliquat 336 and filler in PIM. The value of Δ*H*^0^*_m_* has been estimated to be 104.7 J/g [34]. 

#### 2.3.5. Scanning Electron Microscopy (SEM) Analysis 

The surface morphology of membranes was analyzed by SEM analysis using Carl Zeiss EVO^®^ 40 EP (Germany). Prior to measurements, the samples were subjected to carbon coating deposition.

#### 2.3.6. Particle Size Distribution

The particle size distribution of CNa and C30B was analyzed by a dynamic light scattering (DLS) measurement technique using Zetasizer Nano ZS (Malvern Instruments, Malvern, UK). Samples were diluted in deionized water and the dispersion was submitted to ultrasound excitations in order to avoid the formation of aggregates. A polystyrene cell of 1 mL was used for the measurements. All analyses were performed in triplicate. 

### 2.4. Transport Experiments

Transport experiments were carried out in a glass cell, comprising two removable cylindrical compartments (V = 200 cm^3^). The membrane (diameter: 5 cm) was sandwiched between these two compartments and carefully sealed with poly(tetrafluoroethylene) (PTFE) plane seals. A Cr(VI) solution in 0.1 M HCl was used as the donor phase and the acceptor phase solution was buffered by a solution of acetic acid and ammonium acetate at pH 5. Solution in each compartment was stirred at 500 rpm using magnetic stirrer. The cell was immersed into a thermostatic waterbath for the temperature regulation (25.0 ± 0.1 °C). The samples (0.1 mL) were taken periodically from both compartments with a micropipette and analyzed in an acidic medium using a UV–visible spectrophotometer at 540 nm (Thermo Fisher Evolution 220). 

The kinetic transport parameters were calculated according to Equation (2).

(2) $$\mathrm{Ln}\;(\frac{C_{feed}}{C_{0feed}})=-kt$$

where $C_{feed}$ and $C_{0\;feed}$ is the Cr(VI) concentration in the feed phase at time *t* and the initial Cr(VI) concentration in the feed phase (i.e. at *t* = 0), respectively. 

The relationship Ln ($\frac{C_{t\;feed}}{C_{0\;feed}}$) versus time was linear, that was established by the values of the determination coefficient (R^2^) close to 1 (i.e., 0.999). The permeability coefficient *P* was determined according to Equation (3).

(3) $$P=\frac{V}{A}\;k$$

where *V* is the volume of the aqueous feed solution, and *A* is the active membrane area. The initial flux $J_{i}$ is determined by Equation (4).

(4) $$J_{i}=PC_{i}.$$

## 3. Results and Discussion

### 3.1. Particle Size Distribution

One of the advantages of the clay using in the polymer membranes is their large aspect and interfacial area. Thus, to obtain good macroscopic properties of PIMs as well as improved mechanical, thermal and permeation properties, well dispersion of clays in the matrix polymer is especially desirable. Nevertheless, the total clay exfoliation is rather difficult to achieve and most polymer/clay nanocomposite membranes present regions, in which aggregates and partially intercalated and exfoliated structures coexist. Therefore, prior to the membrane Cr(VI) transport characterization, it is important to analyze the particle size distribution of clay platelets incorporated into a polymer matrix and to determine the mechanical properties and the morphology of the loaded membranes. Vollenberg and Heikens reported that the elastic modulus increased with decreasing particle size for composites based on polysulfone, polycarbonate, and polypropylene and contain glass or alumina particles [35]. On the other hand, the particle size strongly influences the permeation of small molecules through the filled membranes. It is shown that the gas (N_2_, O_2_, and CO_2_) permeability of the polydimethylsiloxane (PDMS)/silicalite mixed matrix membranes increases with the particle size increasing due to the enhanced area and number of the polymer/zeolite interfaces [36]. 

**Figure 2:**
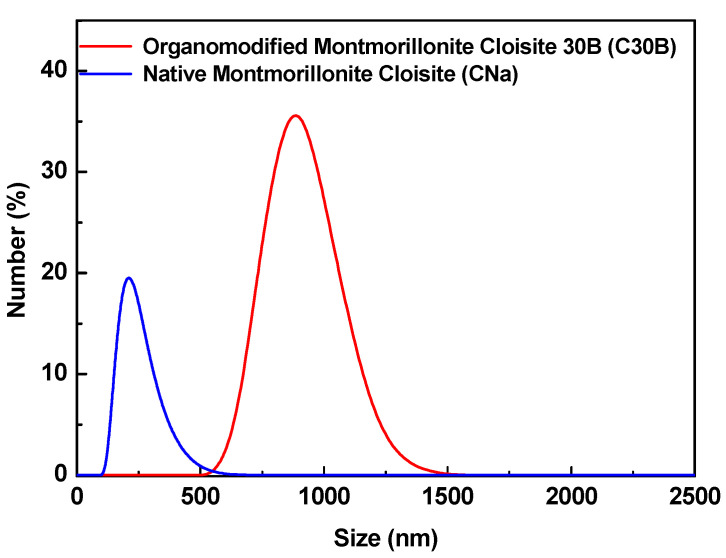
Particle size distribution of natural sodium (CNa) and organo-modified (C30B) MMTs.

The results of the DLS measurements (Figure 2 and Table 1) reveal that the particle size distribution for both types of MMT is monomodal. However, as one can see, both clays (i.e., native (CNa) and organo-modified (C30B) MMTs) are present in their aggregate form as practically 90% of clays have a dimension less than 1.3 µm for C30B and less than 0.5 µm for CNa.

The mean size of the organo-modified MMT is higher than that of the native MMT by a factor of 3 (Table 1). This difference may be explained by the presence of the surfactant in C30B, which increases the inter-layer distance between platelets. Therefore, two different structures for PIMs loaded with CNa or C30B can be obtained with rather distinct permeation behavior.

### 3.2. Membrane Morphology 

It has been already shown that homogeneous nanoparticle dispersion in a polymer matrix makes possible significant improvement of mechanical strength and better controlled transport properties [37,38]. It is shown that the good dispersion of more or less exfoliated fillers in a semi-crystalline polymer (such as PVDF) can hinder its crystallization, while a less successful dispersion (i.e., intercalation and aggregation) can promote the growth of PVDF crystals [39]. It is known that the nanoparticles can have a nucleating effect depending on the particle size, their quantity and compatibility with the polymer, so the polymer crystallinity can be reduced or, on the contrary, increased. In addition, the filler presence can also change the crystalline structure. In the case of PVDF/MMT composite, it is found that the MMT presence is favorable to produce the piezoelectric β phase of PVDF and causes the internal stress in α crystals [40]. Also, the PVDF crystallinity and spherulite size decrease with the MMT content rising. The increase of the melting temperature and induced β phase is also observed for the PVDF composite with functionalized GO [41]. 

**Figure 3:**
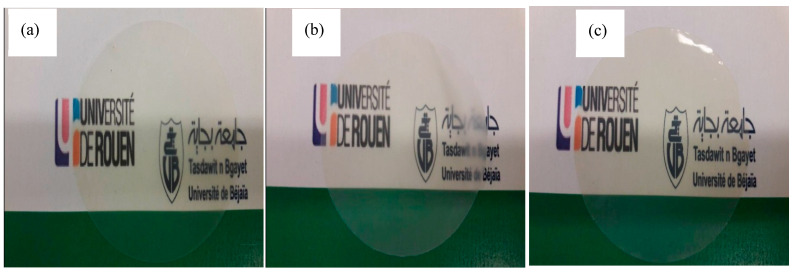
Optical images of the membranes: (**a**) 70PVDF/30Aliquat 336, (**b**) 50PVDF/30Aliquat 336/20CNa, (**c**) 50PVDF/30Aliquat 336/20C30B.

To study the compatibility between the incorporated fillers and the polymer matrix, a visual observation is preliminarily carried out by comparing the loaded and unloaded PIMs. The optical images of PVDF/Aliquat 336 membranes with and without fillers are shown in Figure 3. As one can see, the PVDF membrane with 30 wt % of Aliquat 336 is a transparent film with a homogeneous surface (Figure 3a). After the incorporation of 20 wt % of native MMT (CNa) into the PVDF matrix, the membrane is still transparent with non-oily surface (Figure 3b). This result testifies to the fact that the solid-liquid phase separation does not take place and, so, the ionic liquid (Aliquat 336) is compatible with the mixed PVDF/CNa matrix. The membrane is a little more brittle and a little more translucent than that without filler (compare Figure 3a,b) that may indicate the insufficient dispersion of the filler in the PVDF/Aliquat 336 matrix. However, taking into account the great quantity of CNa particles in the membrane (i.e., 20 wt %), one may suppose that the dispersion is relatively good.

As the native MMT is hydrophilic, it is very difficult to separate the sheets and to disperse them homogeneously within a low polar polymer matrix. Therefore, numerous aggregates and/or intercalated structures of MMTs may be observed in such matrices [42,43]. To overcome this difficulty, it is required to use organo-modified MMT, which is less hydrophilic and is characterized by higher inter-layer distance between clays that favors macromolecular chain diffusion between platelets [44,45]. Thus, in case of PVDF-based membranes, it is more appropriate to use C30B as a filler. Really, the membrane containing 20 wt % of C30B, 30 wt % of Aliquat 336 and 50 wt % of PVDF (Figure 3c) appears to be more transparent and smoother compared to the membrane containing CNa (Figure 3b). Such a result may be explained by a better compatibility between C30B clays and the PVDF chains, thus leading to a better dispersion of nanoclays in the PVDF matrix and probably inducing a reduction of the spherulite formation and, so, a crystallinity decrease [39]. 

**Figure 4:**
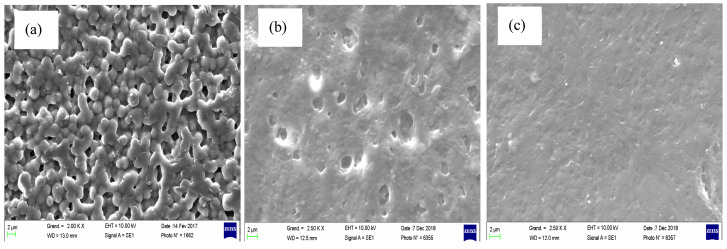
Surface SEM images of different PIMs: (**a**) 70PVDF/30Aliquat 336; (**b**) 50PVDF/30Aliquat 336/20CNa; (**c**) 50PVDF/30Aliquat 336/20C30B.

The PIM structure and morphology are crucial parameters as they strongly influence transport and extraction properties. An asymmetric or porous structure, for example, offers more exchange surface between the membrane and the solution to be treated. To analyze the effect of the filler incorporation on the PIM morphology SEM analysis is performed (Figure 4). As one can see, the unloaded membrane has rough surface containing pores (Figure 4a). The surface roughness is the result of the spherulites growth in the PVDF crystalline phase during the separation of the solid/liquid phase during the solvent evaporation [20]. In the presence of the nanofillers, the membrane surfaces changes (Figure 4b,c). One can clearly observe the disappearance of the granular structures on the membrane surface. This fact can be explained by a reduction of the spherulite formation and, consequently, a decrease of the crystallinity degree in the MMT presence. Thus, the membrane surfaces become more homogeneous, smoother, and less porous, especially in the presence of C30B. Indeed, one can see the absence of macropores in the case of the PIM loaded with C30B (Figure 4c) probably due to a better compatibility between C30B and the PVDF/Aliquat 336 matrix. On the contrary, in the case of the membrane containing CNa (Figure 4b) the presence of more or less deep cavities can be observed. Such a cavity is due to the poor affinity between CNa particles and the hydrophobic PVDF matrix. These pores and pinholes can make the membrane more permeable [20,46]. However, the filler aggregates are not clearly observed on the membrane surface whatever the filler used (i.e., CNa or C30B), confirming a relative homogeneous dispersion of MMT in the PVDF/Aliquat matrix. Because of the lower affinity of CNa with PVDF (in comparison with C30B) it is possible that the distribution of the native fillers is less homogeneous in the membrane and probably these fillers are more numerous close to the membrane surface.

### 3.3. Hydrophobic/Hydrophilic Balance

The membrane hydrophilic/hydrophobic balance plays a determining role in the transport and stability properties of the PIMs. Indeed, if the PIMs with a hydrophilic character reveal improved transport of the targeted species as the hydration process is enhanced in this case [20,21], the membrane hydrophobic character ensures a good PIM stability owing to the carrier loss delay [22,29]. 

**Figure 5:**
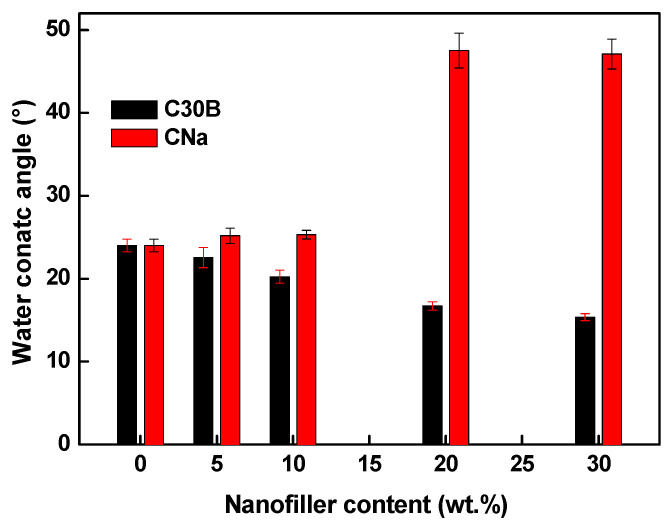
Water contact angle of the PVDF/Aliquat 336 membrane as a function of the filler content. The Aliquat 336 content was fixed at 30 wt % and the PVDF/filler content was varied. The error bars are ± SD (standard deviation).

The water contact angle measurements allow us to study the effect of the filler incorporation on the membrane hydrophilic/hydrophobic behavior. As expected, the mean water contact angle value of the pure PVDF membrane is about 83° confirming its hydrophobic character [47]. After inclusion of Aliquat 336 (30 wt %), the contact angle decreases significantly and reaches ~24° (Figure 5). The presence of quaternary ammonium groups in Aliquat 336 is responsible for this hydrophilic behavior [48]. 

The influence of the filler presence on the hydrophilicity of the PVDF/Aliquat 336-based PIMs is shown in Figure 5. In case of C30B, the contact angle gradually decreases with the rising of the filler quantity. This result indicates that a membrane surface becomes more hydrophilic. Similar behavior is observed for the PIMs based on poly(vinyl chloride) (PVC) and di-(2-ethylhexyl) phosphoric acid (D2EHPA) loaded with silver nanoparticles [49]. Generally, the decrease of the water contact angle in the nanocomposite is attributed to the non-uniform dispersion of the hydrophilic inorganic fillers within the membrane resulting in nanoparticles aggregation on the membrane surface [49,50]. However, in our case the SEM images did not reveal any assembly of nanoparticles on the PIM surface (Figure 4). In fact, in addition to the surface polarity, the membrane surface topography may also influence the water contact angle. In the case of the PIMs loaded with C30B (Figure 4c), the surface topography is much less pronounced as compared with pure PVDF/Aliquat 336 (Figure 4a) and that with CNa particles (Figure 4b). Therefore, the reduced surface roughness and the absence of macropores result in the water contact angle decrease. 

Surprisingly, the presence of native MMT causes a significant increase in the membrane water contact angle when its content exceeds 10 wt %—the angle increases from ~24° (for 70PVDF/30Aliquat 336) to ~47° (for the PIMs with 20 wt % and 30 wt % of CNa) (Figure 5). An increase of the membrane surface hydrophobicity was obtained also in case of the PIM with RGO [29]. In any case, it should be mentioned that the increased hydrophobic character of the PIM containing CNa can enhance the membrane stability without improving the PIM permselective properties.

Since CNa is more hydrophilic than C30B (the latter having hydrophobic alkyl chains), increasing the water contact angle for the PIMs with CNa could be explained by the surface roughness, the pore presence, by the PVDF crystalline state and also by the possible different Aliquat 336 distribution on the membrane surface. Really, the membrane surface may contain less Aliquat 336 in case of the PIM with CNa. However, at this stage of the discussion it is rather difficult to explain more precisely the contribution of the pore presence and surface roughness on the water contact angle increase. It is known that the surface roughness increase as well as the presence of micro-pores can reduce the membrane wettability as it is shown by the models of Wenzel [51] and Cassie-Baxter [52]. 

Due to the low affinity between CNa and Aliquat and also PVDF, it is possible that the amount of Aliquat 336 at the membrane surface is lower for the PIM containing CNa in comparison with the PIM containing C30B which is more hydrophilic. Thus, it can be supposed that when the CNa amount increases (more than 10 wt %), the surface enrichment with CNa leads to a membrane surface mainly composed of the PVDF matrix containing more or less dispersed CNa particles. As expected, the presence of CNa at the membrane surface provokes the decrease of the water contact angle in comparison with pure PVDF (48° and 83°, respectively). This decrease is less than that for the PVDF membrane with Aliquat 336 (24°), confirming the presence of Aliquat 336 on the surface in less extent in the presence of CNa. 

### 3.4. Membrane Mechanical Performance 

**Figure 6:**
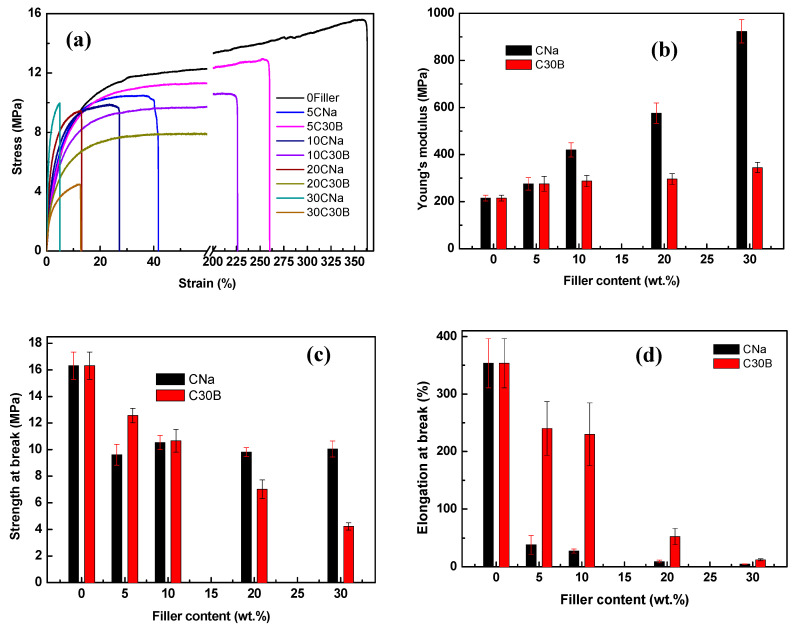
Mechanical properties of the different PIMs: (**a**) stress–strain curves; (**b**) Young’s modulus, (**c**) strain at break, and (**d**) elongation at break. The error bars are ±SD (standard deviation).

Tensile tests were performed in order to determine the PIM mechanical properties. The stress–strain curves and the deduced tensile parameters (i.e., strength at break, elongation at break, and Young’s modulus) of the different membranes are shown in Figure 6. The PVDF membrane has the strength at break value of 42.6 ± 3.2 MPa, elongation at break value of 6.05 ± 0.5% and Young’s modulus equals to 1560 ± 69 MPa. These values confirm the strength and the rigidity of this membrane and are similar to results reported earlier [20]. It should be noted that the mechanical properties of the PVDF membrane depend strongly on the method of its preparation and the used solvent/polymer ratio [53,54]. 

As expected, the addition of Aliquat 336 changes the PVDF mechanical behavior as significantly higher elongation at break (~350%) is obtained due to the plasticizing effect of Aliquat 336 [20]. Such ductile behavior is also reported for the PVDF membranes doped with other ionic liquids, for example 1-butyl-3-methylimidazolium bis(trifluoromethylsulfonyl)imide [34,55]. 

Generally, the incorporation of a small amount of filler (<5%) (for example, carbon nanotubes [56], native or organo-modified clays [57]) to the PVDF membrane leads to its mechanical reinforcement through the reduced elongation at break and increased stress at break and Young’s modulus values. However, when a greater filler amount is added, an opposite effect can be observed. For example, a deterioration of the mechanical properties of the PVDF/hydroxyapatite (HAP) membrane was observed when 30 wt % and 40 wt % of HAP were added [47]. In addition, the mechanical properties of the PIMs developed in this study may be different from those of the conventional nanocomposites (i.e., polymer matrix/filler) as the possible interactions of an ionic liquid with the filler and polymer matrix can modify the nanoparticle dispersion and, therefore, the membrane mechanical properties. Indeed, the mechanical properties of polymer/filler composites are governed by several microstructural parameters, such as the matrix and filler properties, their distribution as well as polymer/filler interfacial adhesion, and by the method of nanocomposite elaboration [58].

As one can see from Figure 6, the addition of CNa or C30B nanofillers influences differently the elastic behavior of the studied PIMs. In case of CNa, a significant decrease of the strength at break (Figure 6c), but especially the elongation at break (Figure 6d) is obtained accompanied by a strong linear increase of the Young’s modulus value (Figure 6b). This result is characteristic to nanocomposites, in which micro/nanoclays play the role of reinforcement as it can be seen by an increase in stiffness (i.e., Young’s modulus) and by the flexibility reduction (i.e., elongation at break). In case of reinforcement, however, one would expect an increase in the strength and mechanical resistance that would lead to a higher tensile strength at break value, which is not the case for these PIMs (Figure 6). This result is due to the hydrophilic nature of CNa fillers that aggregate and are poorly dispersed in the mixed PVDF/Aliquat 336 matrix because of weak interactions between membrane components.

In case of C30B, the slight increase of the Young’s modulus value (Figure 6b) and the great decrease of the tensile strength (Figure 6c) indicate a lack of the interfacial adhesion. However, unlike CNa, the addition of 5 and 10 wt % of C30B allows us to maintain high membrane flexibility (i.e., high elongation at break) and strength at break (Figure 6c,d). This fact can be attributed to a slightly more homogeneous distribution of the C30B clays in the PVDF/Aliquat 336 matrix. Such a result is not surprising as the presence of alkyl chains in silicate particles after the MMT modification makes them less hydrophilic, thus improving their compatibility with the polymer matrix and their dispersion [42,43]. Above 10 wt % of C30B, the membrane mechanical properties are deteriorated due to the important filler aggregation. It should be noted that the increase of Young’s modulus is higher when CNa is incorporated (Figure 6b). This behavior can be explained by the filler size [35], the C30B has a greater size as compared with that of CNa (851 nm and 273 nm, respectively, Table 1). So, the C30B incorporation into the PVDF/Aliquat 336 matrix can create more micro-voids and free volume leading to the decrease of the membrane density and, thus, reducing its stiffness as compared with the PIMs with CNa. If the addition of a high filler amount weakens the elaborated PIMs; however, they remain easy to handle and can be applied in diffusion experiments without the risk of puncturing or breaking. Also, the stiffness rising of the PIMs loaded with CNa platelets is a positive point for their stability as the rigidity may reduce the carrier mobility within the polymer membrane and, therefore, may prevent its leakage into the adjacent aqueous phases during prolonged use.

### 3.5. Membrane Thermal Characterization

**Figure 7:**
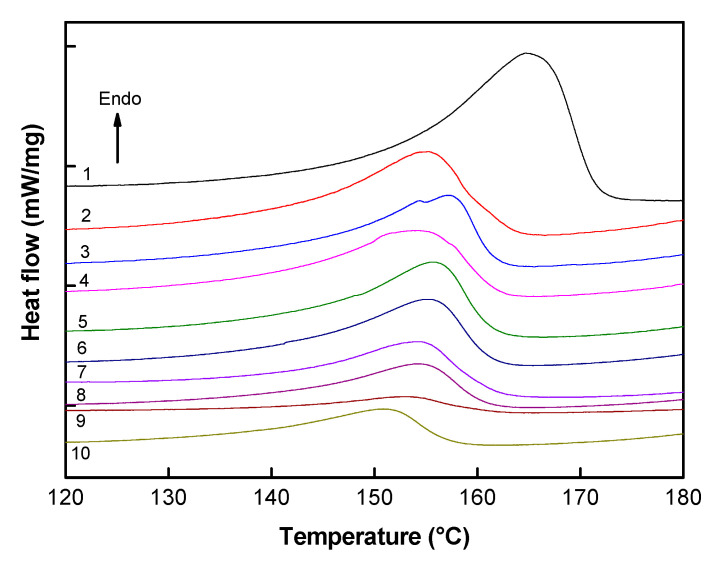
Thermograms of the studied PIMs (first heating scan): (1) PVDF, (2) 70PVDF/30Aliquat 336, (3) 65PVDF/30Aliquat 336/5C30B, (4) 65PVDF/30Aliquat 336/5CNa, (5) 60PVDF/30Aliquat 336/10C30B, (6) 60PVDF/30Aliquat 336/10CNa, (7) 50PVDF/30Aliquat 336/20C30B, (8) 50PVDF/30Aliquat 336/20CNa, (9) 40PVDF/30Aliquat 336/30C30B, (10) 40PVDF/30Aliquat 336/30CNa.

To study the influence of the MMT incorporation on the evolution of the membrane crystallinity, DSC measurements were carried out. The DSC thermograms of the first heating of the studied PIMs are shown in Figure 7 and the obtained thermal characteristics are gathered in Table 2. It can be seen that the melting temperature of the composite membranes is shifted to lower temperatures as compared with the PVDF membrane. This decrease can be explained by the incorporation of the ionic liquid (Aliquat 336) into the PVDF matrix that leads to strong electrostatic interactions between Aliquat 336 and the PVDF polymer chains. A lower thermal energy is, therefore, sufficient for the melting of the crystalline phase [20,55]. The incorporation of the ionic liquid leads also to a decrease in the PVDF crystallinity—50% in case of pure PVDF and 38% in case of the 70PVDF/30Aliquat 336 membrane (Table 2). This result agrees well with the decrease of the melting temperature. The presence of the ionic liquid within the polymer matrix prevents the arrangement of the PVDF macromolecular chains owing to the new interactions between Ammonium chloride and fluorinated chains, thus hindering the PVDF chain crystallization and increasing the amorphous phase percentage [34]. Such behavior is similar to that noted for the PVDF membranes doped with other ionic liquids [20,34]. In addition to Aliquat, the presence of MMT also contributes to the decrease of the PVDF crystallinity (Table 2). However, considering the DSC measurement errors, it is difficult to see some tendency of the influence of the MMT nature on the crystallinity decrease. One can only assume that the C30B clay (with hydrophobic alkyl chains) has more affinity to the hydrophobic PVDF matrix than CNa and, therefore, it can more easily interrupt the PVDF crystallization. Indeed, the PIM containing 30 wt % of C30B has the lowest crystallinity degree of 12% (Table 2). This result is in agreement with the SEM results, showing that the PIM surface is characterized by the absence of spherulites (i.e., surface crystal structure) (Figure 4). The MMT ability to change the PVDF crystallinity has been already reported by Lee et al. [39]. In that case, two types of MMT (C30B and C15A) were incorporated into a PVDF matrix and a decrease of the PVDF crystallinity was observed. Moreover, the nanocomposites with C15A revealed much lower crystallinity than those with C30B, whatever the filler content was. Therefore, one can conclude that the presence of organoclay disturbs the PVDF crystallization and this effect is much stronger if the filler has higher affinity with the polymer. The decrease of the membrane crystallinity should enhance the Cr(VI) transport performance as the solution diffusion occurs only in the po- lymer amorphous phases [2,18,19,20].

**Figure 8:**
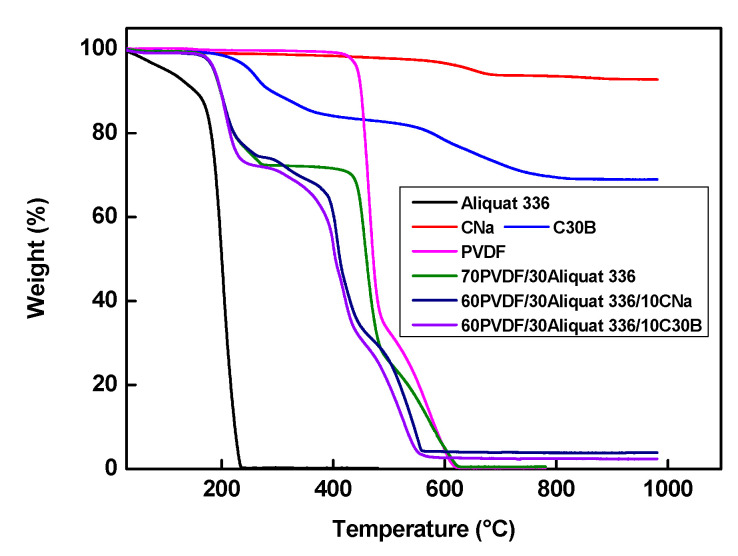
Thermogravimetric curves of elaborated PIMs and their individual components.

In order to study the thermal stability of the obtained membranes, the thermogravimetric analysis was performed (Figure 8). For better comparison, the analysis was also carried out for membrane individual components (i.e., Aliquat 336, CNa, and C30B). As one can see, CNa presents a mass loss of about 8% above 600 °C due to the dehydroxylation of aluminosilicate groups of the silicate structure. C30B starts to degrade at around 200 °C and lost *ca.* 30% at 800 °C. Such weight loss is probably due to the loss of alkyl chains grafted on silicate sheets [59]. As to Aliquat 336, it starts to decompose at 150 °C. The mass loss recorded at the beginning of the heating is linked to the presence of moisture trapped in the sample [20]. 

The degradation process of the pure PVDF membrane takes place in one step at 450 °C owing to the breaking of C-H and C-F bonds and to the formation of C=C bond [20,55]. The thermal behavior of the 70PVDF/30Aliquat 336 membrane is characterized by two degradation steps. The first step starts at 170 °C and is related to the Aliquat 336 degradation; and the second step observed at 430 °C relates to the degradation of the PVDF ske-leton (Figure 8). It should be point out that the incorporation of Aliquat 336 into the PVDF matrix delays the degradation of the ionic liquid due to its physical immobilization in the PVDF matrix [20]. 

The MMT presence has no influence of the PIM thermal stability since the onset degradation temperature of the loaded PIMs is practically the same as that for the unloaded PIM whatever the MMT type is (i.e., CNa or C30B). Considering this result, one can conclude that the elaborated PIMs are stable up to 170 °C. Such thermal stability is sufficient for the membrane use in the separation process at high temperatures without any risk of the membrane degradation or deterioration.

### 3.6. Membrane Transport Properties 

The transport process through the PIM consists in the ion exchange on both membrane/solution interfaces. At operational pH (i.e., pH = 1), the HCrO_4_^−^ ion species predominate in the aqueous donor medium [20]. Therefore, at the beginning, the target species are bound by the extractant at the feed solution/membrane interface. Then, the formed metal/ion carrier pair diffuses through the PIM under a driving force (chemical potential) from the feed solution/membrane interface to the membrane/acceptor phase interface. After this step, the formed metal/ion carrier pair can be stripped (i.e., back-extracted) into a receiving aqueous solution by a suitable stripping reagent (e.g., NaOH) [3].

Therefore, the transport properties of the elaborated PIMs of different composition were studied. The influence of the filler and its content on the Cr(VI) transport performance was examined. In addition, the PIM stability was also evaluated. For reproducibility, the measurements were repeated at least three times for the membranes prepared from the same casting solution as well as for the membranes prepared with different casting solution. Prior to measurements of the loaded PVDF-based PIMs, the pure PVDF membrane and the PVDF membrane containing only the filler (i.e., C30B or CNa) were tested. However, in the absence of the ion carrier (i.e., Aliquat 336), no transport from the donor to acceptor phase was detected. This result confirms that the membrane without the ion carrier acts as a barrier to the metal ion permeation and, so, the presence of the carrier agent in the PIM is essential to the Cr(VI) extraction.

#### 3.6.1. Influence of the Filler Nature and Its Content 

There are numerous studies describing facilitated transport across the PIMs [13,21], however, the facilitated transport through the PIMs loaded with nanoclays is rather new subject and only few articles that can be found in literature [29,30,49,60,61]. Besides, no correlation between the structure of the PIMs containing nanoparticles and their transport performance exists. 

**Figure 9:**
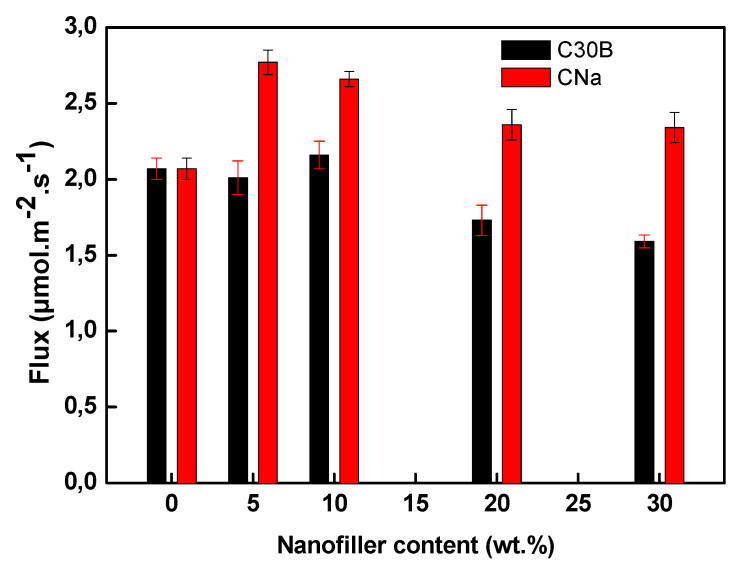
Variation of the Cr(VI) flux through studied PVDF/Aliquat 336 PIMs. Membrane thickness: 40 ± 3 µm. Feed phase: Cr(VI) (10 mg/L) in 0.1M HCl, acceptor phase: acetic acid and ammonium acetate buffer at pH 5. Duration of experience: 8h. Stirring speed: 500 rpm. The error bars are ±SD (standard deviation).

The transport performance of Cr(VI) ions through the elaborated membranes based on PVDF and Aliquat 336 (30 wt %) used as ion carrier is shown in Figure 9 as a function on the MMT type (CNa or C30B). The obtained results are gathered in Table 3. As one can see, the permeation flux of Cr(VI) through the 70PVDF/30Aliquat 336 membrane is 2.07 ± 0.07 µmol/(m^2^·s). This value is similar to that recorded by Turgut et al. in case of the Cr(VI) removal using a membrane based on PVDF-*co*-hexafluoropropylene (HFP) containing 30 wt % of 1,3-didecyl-1H-imidazol-3-ium bromide [62].

The addition of the CNa filler to the PVDF/Aliquat 336 membrane increased the permeation flux up to 2.7 µmol/(m^2^·s) and 2.6 µmol/(m^2^·s) for the PIMs containing 5 wt % and 10 wt % of CNa, respectively (Figure 9 and Table 3). Such improvement of the permeation flux is related to increased surface roughness and the presence of more free volume (microvoids) and surface pores (Figure 3) as well as to the increasing of the PVDF amorphous phase percentage (Table 2). Kaya et al. noted an improvement of the Cr(VI) flux through a CTA-based membrane containing 1 wt % of RGO as compared with the same membrane but without RGO owing to the membrane modified structure with higher roughness [29]. Maiphetlho et al. also reported an increase in the permeability of Co(II), Cd(II), Cu(II), and Ni(II) ions through a membrane composed of PVC and D2EHPA containing 10 wt % of silver nanoparticles [49]. The authors attributed this improvement to a homogenous distribution of silver nanoparticles within the membrane and the membrane high water uptake. Other authors also attributed the improvement of the transport performance to the higher surface area and anion exchange capability of filler [61], and to the affinity of incorporated nanoparticles with target species [38], which results in the improved membrane selectivity [30,49,61]. 

In the case of C30B, no significant change in the Cr(VI) flux is observed up to 10 wt % (Figure 9 and Table 3). Indeed, the addition of C30B results in a PIM with a smooth and more dense structure (Figure 3). Moreover, the membrane wettability is not modified very much in this case (Figure 5) meaning that the clay presence (≤10 wt %) has practically no influence on the tortuosity and, thus, the same Cr(VI) permeation flux is obtained (Figure 9).

However, above 10 wt % of CNa and C30B, a decrease in the Cr(VI) permeability is observed, the reduction being higher in case of C30B (Figure 9). In both cases, the decrease of the Cr(VI) flux through the filled PIM can be attributed in part to the increase of the membrane stiffness and its ductile behavior, which is strongly decreased when the filler concentration is above 10 wt % (Figure 6b,d), thus provoking a reduced mobility of the Aliquat 336/Cr(VI) complex. O’Bryan et al. revealed that the increase of the PIM rigidity may decrease the mobility of the carrier molecules resulting in decreasing the membrane permeability [24]. Besides, the addition of nanoparticles to the membrane composition leads to an increase in the membrane crystalline regions and, therefore, the increased Tortuosity allows extending the diffusion path of the Aliquat 336/Cr(VI) complex and, so, the permeation decreases [60]. Moreover, the high filler concentration in a membrane results in a non-homogeneous filler dispersion within the polymer matrix that causes a decline in the membrane properties [49]. The more hydrophobic membrane nature in the presence of high concentration of CNa (Figure 5) may also contribute to the Cr(VI) flux reduction. The similar results were obtained for different PIMs as the membrane transport efficiency was deteriorated with the high (>10 wt %) filler concentration owing to the increased polymer crystallinity [49,60,61]. In our case, in addition to the increased stiffness (Figure 6), the Cr(VI) permeation decrease can be also explained by the increased tortuosity induced by the clay presence with high concentration. 

#### 3.6.2. Membrane Stability and Reusability

**Figure 10:**
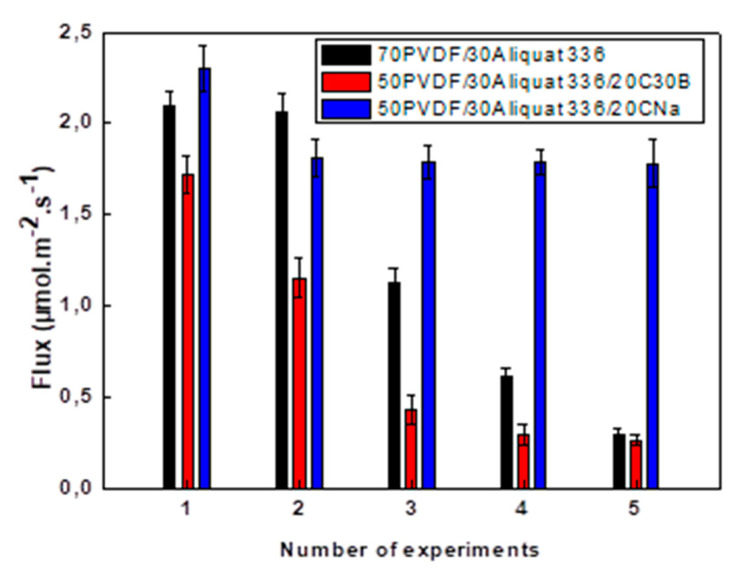
Cr(VI) flux evolution as a function of the number of measurements for the same membrane. Membrane thickness: 40 ± 3 µm. Feed phase: Cr (VI) (10 mg/L) in 0.1M HCl, acceptor phase: acetic acid and ammonium acetate buffer at pH 5. Duration of one cycle: 8 h. Stirring speed: 500 rpm. The error bars are ± SD (standard deviation).

As mentioned previously, the filler incorporation into the PIMs aims to improve their stability and repeated use, which are key factors determining the PIM efficiency and viability in analytical, environmental, and industrial separation [21]. Considerable efforts have been made to improve the PIM stability. However, to the best of our knowledge, only few studies focused on the influence of the filler incorporation into PIMs on their stability [29,30,49]. Thus, in the present work the stability of PIMs containing CNa or C30B fillers is analyzed based on the membrane reuse. Repeated transport experiments were performed with the same membrane by replacing the feed and acceptor solutions at the end of each cycle. The evolution of the Cr(VI) flux for each extraction experiment during five cycles for the 70PVDF/30Aliquat 336, 50PVDF/30Aliquat 336/20C30B, and 50PVDF/30Aliquat 336/20CNa membranes is shown in Figure 10. One can see that the studied PIMs exhibit different behavior during prolonged use. The PIM without filler (i.e., 70PVDF/30Aliquat 336) preserves the Cr(VI) flux during two first cycles. However, starting from the third cycle its performance deteriorates and the flux gradually decreases up to 0.3 µmol/(m^2^·s) after 5 cycles. Similar deterioration of the membrane transport properties was observed for the CTA-based PIM [2].

It can be seen that the C30B incorporation does not contribute to the stabilization of the PVDF/Aliquat 336 membranes as a significant decrease of the Cr(VI) flux is observed already starting from the second cycle (Figure 10 and Table 4). However, the fact that the Cr(VI) permeation flux remains lower than that of the unfilled PIM may be explained by the more dense membrane surface and also by the possible interactions of the quaternary ammonium groups of the surfactant of C30B and Aliquat 336. This better affinity can improve the diffusion pathways for Cr(VI) according to a percolation phenomenon induced by the fillers more or less dispersed within the membrane. A similar effect was observed in case of the toluene permeation through polyamide-12 membrane containing C30B particles [63]. The surface tension of Aliquat 336 equals to 0.028 J/m^2^ is very close to that of C30B (0.034 J/m^2^), whereas the surface tension of native MMT is 0.205 J/m^2^ [57]. Indeed, both Aliquat 336 and C30B surfactant contain long alkyl chains, thus explaining their chemical affinity. Therefore, one may conclude that the higher mass decrease in case of the PIM containing C30B is caused also by the simultaneous release of Aliquat 336 (carrier) and C30B. This decrease is favored by the membrane flexibility as the stiffness of the PIM with the C30B filler is much lower in comparison with the PIM with the CNa particles.

On the contrary, a better membrane stability is observed for the PIM containing 20 wt % of CNa (Figure 10 and Table 4). In this case, the Cr(VI) flux decreases slightly from 2.3 µmol/(m^2^·s) to 1.8 µmol/(m^2^·s) and then remains stable up to five cycles. Therefore, one can conclude that the PIM filled with CNa exhibits acceptable stability from the second permeation cycle. The same trend of the membrane stability was reported by O’Bryan et al. for PVDF-*co*-HFP membranes containing 30 wt % of Aliquat 336 [23]. In that case the relative membrane stability was recorded after the thiocyanate extraction efficiency loss after the first cycle. 

The improved stability obtained for the PIMs with CNa in comparison with other membranes can be attributed to the much greater rigidity induced by the clay incorporation—the Young’s modulus values are 576, 296, and 215 MPa for the PIM with CNa, the PIM with C30B and the unloaded PIM, respectively (Figure 6b). Besides, the PIM stability depends strongly on the carrier leaching. This leaching is governed by the carrier mobility within the PIM structure, that is related to the tortuosity of the diffusion paths in the base polymer as well as to the membrane stiffness [24]. In addition, the membrane containing CNa is much more hydrophobic than that without filler or containing C30B (Figure 5). This hydrophobic behavior promotes the membrane stability by delaying the Aliquat 336 leakage in the aqueous phases [22,29]. Due to the incompatibility of CNa with PVDF and its low affinity with Aliquat 336, it is possible that the hydrophilic CNa nanofillers are more concentrated close to the membrane surface while the C30B particles are homogeneously distributed within the matrix. The enrichment of the membrane surface by the CNa nanofillers can reduce the loss of Aliquat 336 from the PVDF membrane.

Kaya et al. reported a better stability for the PIM containing RGO as compared with the unloaded PIM due to the RGO hydrophobic properties [29]. Anticó et al. also noted a better stability of the PIM based on CTA/Aliquat 336 doped with MWCNTs in comparison with the undoped CTA/Aliquat 336 membrane [30]. Such improved stability was explained by the MWCNT ability to adsorb organic compounds, in particular, Aliquat 336.

**Figure 11:**
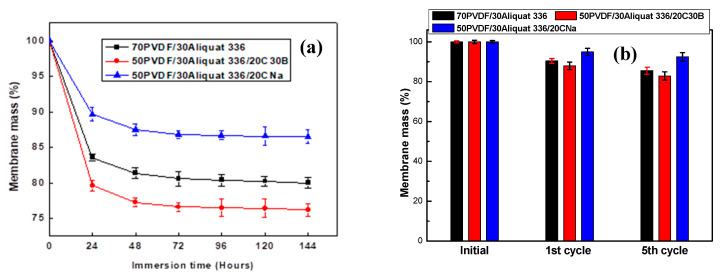
Membrane mass change as a function of the immersion time (**a**) and as a function of the number of measurement (**b**). The error bars are ± SD (standard deviation).

Taking all foregoing into account, a smaller Aliquat 336 leakage into the aqueous phases is expected in the case of the PIM with CNa as compared with other membranes. To verify this, the membrane mass before and after its immersion in deionized water was measured [27,28]. The membrane mass change as a function of the immersion time is shown in Figure 11a. It can be seen that, after the membrane immersion in deionized water, its mass decreases during the first 24 h because of the carrier loss and then the plateau value is obtained whatever the membrane composition is. However, the mass loss depends on the filler nature. The PIM containing CNa exhibits a lower mass loss than other studied membranes according to the following order:

50PVDF/30Aliquat 336/20CNa < 70PVDF/30Aliquat 336 < 50PVDF/30Aliquat 336/20C30B.

Such trend can be explained by the membrane rigidity characterized by the Young’s modulus value (Figure 6b). A slight increase of the Young’s modulus value is obtained for the PIM with the C30B filler (215.0 ± 12.5 MPa for 70PVDF/30Aliquat 336 and 295.9 ± 22.4 MPa for 50PVDF/30Aliquat 336/20C30B). Thus, a higher leakage of the carrier is observed for this membrane owing to the presence of the micro-voids. On the other hand, the high stability of the PIM with CNa may be explained by its high Young’s modulus—almost three times higher than that of the unloaded membrane (215.0 ± 12.5 MPa for 70PVDF/30Aliquat 336 and 575.9 ± 43.3 MPa for 50PVDF/30Aliquat 336/20CNa).

In order to estimate the exact released quantity during the transport process, the PIMs were weighted before and after each cycle (Figure 11b). It is assumed that the mass of the fresh membrane (i.e., before use) is 100% and that the mass loss is due to the Aliquat 336 leakage. One can see that the quantity of the released carrier depends on the membrane composition. Only ~5% of the membrane mass is lost during the first cycle in case of the PIM containing CNa and then the membrane mass remains relatively stable, i.e. ~5% and 7% after the first and fifth cycle, respectively. This result agrees well with the result of the membrane stability (Figure 10) as the membrane mass stabilization is observed after the first cycle. As to the other membranes, a mass loss of 10% and 13% is recorded for the 70PVDF/30Aliquat 336 and 50PVDF/30Aliquat 336/20C30B membranes, respectively (Figure 11b). This loss increases up to 15% and 19%, respectively, with the rise of the number of cycles. Such significant loss of the organic phase explains the deterioration of the membrane stability (Figure 10). 

Therefore, the obtained results confirm that the loss of the extractant is linked with the membrane rigidity, i.e., more the membrane is rigid, more the extractant mobility is reduced and more membrane stability increases.

## 4. Conclusions 

PVDF-based PIMs containing Aliquat 336 as carrier extractant and filled with different types of MMT clays were elaborated by the solvent evaporation technique and used for the removal of toxic Cr(VI) ions. The composition of the loaded PIMs was optimized in terms of the filler nature (native CNa or organo-modified C30B) and its content. The obtained composite membranes were investigated to establish the relationship between the induced structural and morphological changes and the resulting transport properties and their stability performance. 

It was shown that the addition of fillers (CNa or C30B) reduced the PVDF crystallinity. Also, it was observed that the membrane surface was modified by the filler presence as a rough surface was obtained in case of the CNa addition while a smooth and dense surface was obtained with C30B. Tensile tests showed difference in the membrane mechanical behavior according to the filler type. An important increase of the membrane stiffness was noted after the CNa incorporation. Surprisingly, an increase of the hydrophobic character of the mixed PVDF/Aliquat336 membrane was revealed after loading with the more hydrophilic filler, i.e., CNa. All these results allowed us to explain the facilitated transport of Cr(VI) ions through the composite PVDF/Aliquat 336/MMT membranes. 

The obtained composite membranes were used for the facilitated transport of Cr(VI) ions. The membrane containing CNa exhibited better performance with higher Cr(VI) permeability due to its porous structure. This membrane exhibited acceptable stability during five cycles of the Cr(VI) transport experiments, whereas the membranes without filler and those filled with C30B lost their performance in terms of the permeation flux already after the first cycle. Moreover, it is shown that the membrane stability highly depends on the membrane rigidity, since a good retention of the liquid organic phase is observed in case of the more rigid membrane (i.e., the PIM loaded with CNa). Also, owing to the incompatibility of the hydrophilic CNa clay with the hydrophobic mixed matrix PVDF/Aliquat 336, it is possible that the Aliquat 336 distribution is less homogeneous in this membrane in comparison with the PIM loaded with C30B. This fact leads to a PIM with a CNa higher content on the membrane surface, thus reducing the Aliquat 336 leakage. The PVDF/Aliquat 336-based PIMs containing CNa as reinforced particles are promising separator membranes for the Cr(VI) removal in aqueous solutions. Therefore, in future it will be interesting to study these membranes in the selective extraction of Cr(VI) in the presence of other metal ions or in real wastewaters. Also, in order to improve the stability and selectivity of the PIMs, poly(ionic liquids) could be used as ion carrier. The immobilization of such a carrier on the CNa surface could also lead to the decrease of the carrier release from the PIM into the aqueous phase during the prolonged use.

## Figures and Tables

**Table 1:** Particle size distribution of used fillers.

Type of MMT	Average Diameter (nm ± SD)	Polydispersity Index
CNa	273 ± 12	0.348 ± 0.008
C30B	851 ± 35	0.840 ± 0.010

**Table 2:** Thermal characteristics of the elaborated PIMs.

Membrane	Melting Temperature (°C)	Melting Enthalpy Δ*H_m_* (mW/mg)	Crystallinity Degree *X_c_*_(PVDF)_ (%)
PVDF	166.9	51.9	50
70PVDF/30Aliquat 336	155.2	28.1	38
65PVDF/30Aliquat 336/5CNa	154.2	23.7	34
65PVDF/30Aliquat 336/5C30B	157.1	22.9	34
60PVDF/30Aliquat 336/10CNa	155.3	21.6	34
60PVDF/30Aliquat 336/10C30B	155.7	23.2	37
50PVDF/30Aliquat 336/20CNa	154.2	14.1	27
50PVDF/30Aliquat 336/20C30B	154.5	17.1	33
40PVDF/30Aliquat 336/30CNa	150.8	11.0	26
40PVDF/30Aliquat 336/30C30B	153.1	5.0	12

**Table 3:** Variation of the Cr(VI) flux through the elaborated PIMs.

Membrane	Flux (µmol/(m^2^·s))
70PVDF/30Aliquat 336	2.07 ± 0.07
65PVDF/30Aliquat 336/5CNa	2.77 ± 0.08
65PVDF/30Aliquat 336/5C30B	2.01 ± 0.11
60PVDF/30Aliquat 336/10CNa	2.66 ± 0.05
60PVDF/30Aliquat 336/10C30B	2.16 ± 0.09
50PVDF/30Aliquat 336/20CNa	2.36 ± 0.10
50PVDF/30Aliquat 336/20C30B	1.73 ± 0.10
40PVDF/30Aliquat 336/30CNa	2.34 ± 0.10
40PVDF/30Aliquat 336/30C30B	1.59 ± 0.04

**Table 4:** Evaluation of the Cr(VI) flux as a function of the number of measurements.

Number of Cycles	Flux (µmol/(m^2^·s))		
	70PVDF/30Aliquat 336	50PVDF/30Aliquat 336/20CNa	50PVDF/30Aliquat 336/20C30B
1	2.09 ± 0.09	2.30 ± 0.13	1.72 ± 0.10
2	2.06 ± 0.10	1.81 ± 0.10	1.15 ± 0.11
3	1.13 ± 0.07	1.79 ± 0.09	0.43 ± 0.08
4	0.62 ± 0.04	1.79 ± 0.07	0.29 ± 0.06
5	0.29 ± 0.06	1.78 ± 0.13	0.26 ± 0.05

## Data Availability

The data are contained within the article.
